# Transcranial direct current stimulation (tDCS) as an intervention to improve empathic abilities and reduce violent behavior in forensic offenders: study protocol for a randomized controlled trial

**DOI:** 10.1186/s13063-020-4074-0

**Published:** 2020-03-13

**Authors:** Carmen S. Sergiou, Adam J. Woods, Ingmar H. A. Franken, Josanne D. M. van Dongen

**Affiliations:** 1 Department of Psychology, Education and Child Studies, Erasmus University Rotterdam, P.O. Box 1738, 3000 DR Rotterdam, the Netherlands; 2 Center for Cognitive Aging and Memory, McKnight Brain Institute, Department of Clinical and Health Psychology, College of Public Health and Health Professions, University of Florida, Gainesville, FL USA

**Keywords:** Transcranial direct current stimulation (tDCS), Empathy, Violent behavior, Substance use, Forensic offenders, Recidivism, Effectiveness, Ventromedial prefrontal cortex (vmPFC)

## Abstract

**Background:**

Recent studies show that changes in one of the brain areas related to empathic abilities (i.e. the ventromedial prefrontal cortex (vmPFC)) plays an important role in violent behavior in abusers of alcohol and cocaine. According to the models of James Blair, empathy is a potential inhibitor of violent behavior. Individuals with less empathic abilities may be less susceptible and motivated to inhibit violent behavior, which causes a higher risk of violence. Recent neuroscientific research shows that modulating (stimulation or inhibition) certain brain areas could be a promising new intervention for substance abuse and to reduce violent behavior, such as the neurostimulation technique transcranial direct current stimulation (tDCS). This study aims to investigate tDCS as an intervention to increase empathic abilities and reduce violent behavior in forensic substance use offenders.

**Methods/design:**

A total sample of 50 male forensic substance abuse patients (25 active and 25 sham stimulation) will be tested in a double-blind placebo-controlled study, from which half of the patients will receive an active stimulation plus treatment as usual (TAU) and the other half will receive sham stimulation (placebo) plus TAU. The patients in the active condition will receive multichannel tDCS targeting the bilateral vmPFC two times a day for 20 min for five consecutive days. Before and after the stimulation period, the patients will complete self-report measurements, perform the Point Subtraction Aggression Paradigm (PSAP) and a passive viewing empathy task. Resting state electroencephalography (rsEEG) will be performed before and after the treatment period. A follow up will be conducted after 6 months. The primary outcome is to investigate multichannel tDCS as a new intervention to increase empathic abilities and reduce violent behavior in offenders with substance abuse problems. In addition, we will determine whether electrophysiological responses in the brain are affected by the tDCS intervention. Finally, the effects of tDCS on reducing craving will be investigated.

**Discussion:**

This study is one of the first studies using multichannel tDCS targeting the vmPFC in a forensic sample. This study will explore the opportunities to introduce a new intervention to improve empathic abilities and reduce violence in forensic substance use offenders. Specifically, this study may give insight into how to implement the tDCS intervention in the setting of daily clinical practice in this complex, multiple-problem target group and with that contribute to reduction of recidivism.

**Trial registration:**

Dutch Trial Register, NTR7701. Registered on 12 January 2019. Prospectively registered before the recruitment phase. https://www.trialregister.nl/trial/7459.

Recruitment started on the 1st of February 2019 and will be finished approximately in the winter of 2019. Protocol version 1. 22 May 2019.

## Background

Repeatedly using substances has been found to lead to neuro-adaptations in the ventral striatum and ventral tegmental areas and with that, decreased dopamine secretion [1]. Decreased dopamine secretion leads to a higher craving for substances and increased saliency for addictive cues [2–4]. Impaired functioning of the dorsolateral prefrontal cortex (DLPFC) in patients with substance abuse disorders (SUDs) underlies diminished cognitive and inhibitory control and increases the tendency to relapse and maintain addictive behaviors [5–13]. Recent studies show that changes in the brain areas related to less empathic ability (i.e. the ventromedial prefrontal cortex (vmPFC)) in abusers of alcohol and cocaine plays an important role in violent behavior [14, 15]. Preller and colleagues found in their study [15] that cocaine users have deficits in emotional empathy and that patients with substance abuse disorders are less emotionally responsive to the emotions of other individuals and their mental state.

Empathy is crucial for social enhancement, social interactions and relationships, and our emotional and social life [16]. A deficit in empathic ability could lead to antisocial and deviant behavior and with that a higher risk of aggression [15]. Aggression is stated here as behavior that is mostly defined by any behavior that is intended to harm someone who is motivated to avoid being harmed [17]. As said before, antisocial behavior, especially aggression, is associated with dysfunctions in the prefrontal cortex [18–24]. In addition, research has highlighted the importance of executive functions - the “higher” cognitive functions that are controlled by the prefrontal cortex in control of aggression [25–28]. According to the models of James Blair [28–30] violent behavior is inhibited by empathy. Individuals with less empathic abilities may be less susceptible and motivated to inhibit violent behavior, which increases the risk of violence.

### The role of the prefrontal cortex

Several studies show evidence that impaired prefrontal cortex areas lead to the emergence of aggressive behavior. Most notable are impairments in the vmPFC (e.g. emotion regulation, moral decision-making), and the DLPFC (e.g. disinhibition and impulsiveness), which is associated with aggression and violent behavior [25–28]. These dysfunctional prefrontal cortex areas induce psychopathic traits such as blunted emotions and lack of empathy [31] and impaired perspective with increased egocentrism and rigidity [19, 32, 33]. Research has found that children with psychopathic traits have abnormal activity in the vmPFC during a response-reversal task in comparison to children without these traits (Finger et al., 2008). In addition, Raine and colleagues [34, 35] demonstrated the relationship between violence and empathy and argued that abnormal brain structure in the vmPFC causes the most extreme form of empathic inability named “psychopathic predatory violence”. This psychopathic predatory violence correlates with the lack of empathy, impaired moral judgment [34], and hyper metabolism [35] that is associated with increased aggressive impulses.

**Fig. 1:**
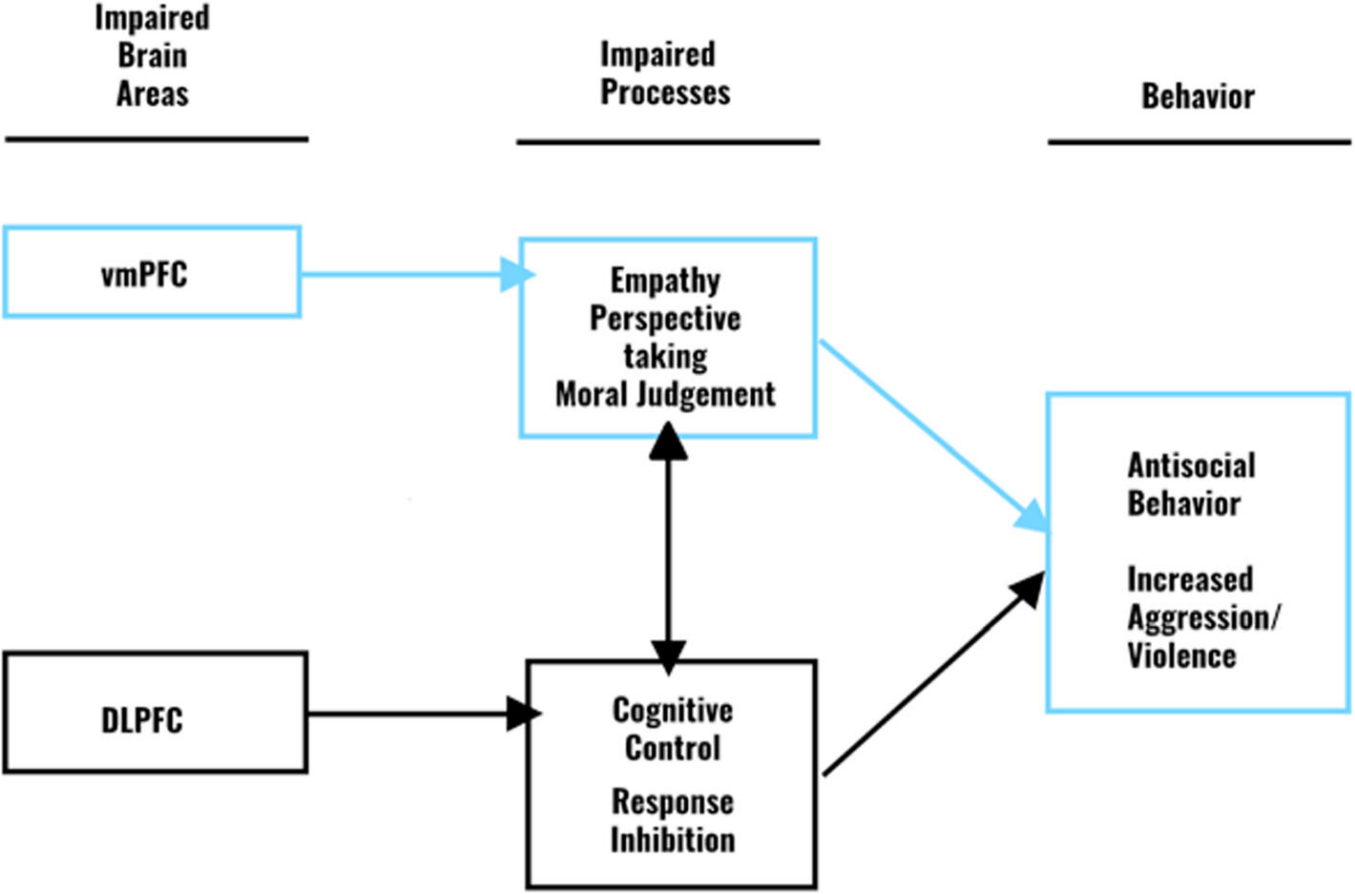
Relationship between the impaired brain areas, impaired processes and behavior. The highlighted brain area, impaired process and behavior are discussed in this paper. DLPFC, dorsolateral prefrontal cortex, vmPFC, ventromedial prefrontal cortex

Figure 1 shows the hypothesized relationship between the impaired brain areas and the subsequent deficits in empathy and violent behavior. Although, the DLPFC modulates the cognitive control and response inhibition that are associated with aggression and violent behavior [25–28], in this study we focus on the vmPFC.

### Ventromedial prefrontal cortex

The vmPFC is particularly relevant to empathic abilities and antisocial behavior [29, 36, 37]. Studies using functional neuroimaging (i.e. functional magnetic resonance imaging (fMRI)) have shown that neural activity in the vmPFC predicts empathic abilities and altruistic motivation [38, 39]. Another longitudinal study [19] demonstrated that lesions damaging the vmPFC, occurring in the first 16 months of human life, result in lifelong psychopathic antisocial traits and will also lead to impaired social and moral reasoning. In addition, damage to the vmPFC is associated with poor decision-making in antisocial behavior [40, 41].

Furthermore, a recent study [42] combining tDCS with fMRI demonstrated that anodal tDCS placed on the forehead led to increased vmPFC activity and decreased negative emotions. Taken together, these studies suggest a potential link between vmPFC functioning and anger regulation.

### Transcranial direct current stimulation

tDCS is a non-invasive neuromodulation technique that modulates the brain region of interest by increasing or decreasing neuronal excitability through constant, low-direct-current electrodes. tDCS has been proven to be an effective intervention to modify brain activity [43–45] and has been investigated in many different disorders [46–48].

In this current study, the effectiveness of tDCS as a new intervention to increase empathy and reduce violent behavior in substance abuse offenders will be investigated. Through modulating (stimulating and inhibiting) certain areas of the brain, tDCS causes a change in the function of the brain, due to an increase in susceptibility to generate and facilitate brain-related electrical impulses. This susceptibility is achieved through repeated sessions of brain stimulation and is thought to produce long term potentiation (LTP)-like “learning” in stimulated neurons. Functional alterations in the brain due to long-term substance abuse are hypothesized to improve with the application of tDCS [49–61] and to reduce craving.

Several studies showed that emotional processes can be influenced through anodal tDCS of the prefrontal cortex (PFC). tDCS can modulate emotional pain [62, 63] and enhance empathy to pain [63]. Interestingly, studies [64–66] suggest that anodal tDCS of the PFC can enhance empathy and increase the feeling of morality. Although there seems to be a clear association between empathy and vmPFC function, only a few studies have investigated modulation of the vmPFC. Abend and colleagues [67] show in their study combining tDCS and fMRI that stimulating with tDCS demonstrated increased emotion-related activation in the vmPFC. Two studies show that anodal stimulation of the (right) vmPFC increases empathic ability and morality [66, 67]. Furthermore, the study of Gilam and colleagues [42] used tDCS simultaneously with fMRI and demonstrated that activity of the vmPFC was increased during active compared to sham stimulation.

In addition to empathy, antisocial behavior, including aggression, is also associated with dysfunctions in the prefrontal cortex [18–24]. Research shows that studies targeting the PFC with anodal tDCS can reduce social exclusion, and the aggressive behavior that emerges from this exclusion [68] can reduce unprovoked aggressive behavior [69] and reduce the intentions of aggressive behavior [70, 71]. Based on the aforementioned theory of Blair linking impaired empathic abilities to violence, and the model that was demonstrated (Fig. 1), it could be proposed that the vmPFC plays a crucial role in modulating both empathic abilities and thereby also in violent behavior. The aforementioned study of Gilam and colleagues [42] also demonstrates the relationship between the vmPFC and violent behavior and shows a decrease in violent behavior in the first session after active tDCS.

In addition to aberrant functioning of specific brain areas, recent research [72, 73] also shows that functional connectivity of brain areas is also affected in individuals with less empathic abilities. Therefore, it is important for the evaluation of the effectiveness of a tDCS intervention, to determine how the resting-state connectivity of the patients, measured with resting state electroencephalography (rsEEG), changes from pre-test to post-test with the intervention. Prior research demonstrates that tDCS can alter functional connectivity and that connectivity change is related to the treatment response (i.e. effectiveness) [74]. To optimize the intervention and increase current focality we use a newer development of the original. This technique, called high-definition tDCS (HD-tDCS or multichannel tDCS) uses gel-based electrodes similar to those used in EEG [75] and is more precise in targeting the brain area of interest. Therefore, in this study we will investigate the idea that multichannel tDCS applied over the vmPFC will increase empathic abilities and subsequently reduce the risk of violence.

#### Aims of the study

The aim of this study is to investigate the effectiveness of tDCS in increasing empathy and reducing violent behavior in offenders with substance abuse problems.

The following research questions will be addressed in this study:

1. Does stimulation with multichannel tDCS targeting the vmPFC increase empathic abilities in forensic patients with substance abuse problems during an empathy task from pre intervention to post intervention?
2. Does stimulation with multichannel tDCS targeting the vmPFC reduce aggressive response and risk of violence risk in patients with forensic substance abuse from pre intervention to post intervention?
3. Does multichannel tDCS targeting the vmPFC reduce craving in patients with forensic substance abuse?
4. Does multichannel tDCS targeting the vmPFC affect electrophysiological response in the brain measured through EEG from pre intervention to post intervention?

Outcomes will contribute to the development of more effective diagnostics and treatment of patients with substance abuse. Furthermore, these data will potentially contribute to improving treatment through increasing understanding of specific targets for treatment interventions. In addition, outcomes may provide better insight in the functioning of the vmPFC and the relationship between empathy and risk of violence.

## Methods/design

### Setting

The study will be carried out in two forensic institutions. The sample will be recruited from the *Forensiche Verslavings Kliniek* (FVK), the forensic addiction clinic of Bouman, Antes. The institution is located in Rotterdam, The Netherlands. The sample size is based on other studies; we will operate according to the “Evidence-based guidelines on the therapeutic use of transcranial direct current stimulation (tDCS)” published in 2017 [76], and seen as achievable, due to the fact that the applicant is currently obtaining research at FVK Bouman Antes, which will make the inclusion of the patients more feasible.

### Procedure/design

In this double blind, placebo-controlled study, a total of 50 male participants between the age of 18 and 60 years will be randomly assigned to either the active condition or the sham condition. Eligible participants will be given written and verbal information about the study and will be invited to participate. After providing informed consent they will participate at the forensic clinic where they are admitted and all the data collected will be anonymous and linked to their participant number.

#### Blinding and randomization

Participants and investigators are blind to the tDCS condition allocation. An external researcher is the only one who knows which participant number corresponds with each condition. The principal investigator, the patients and the research assistants do not know which condition is being executed. The trial established procedures to maintain separation in knowledge between the head researcher and the principal investigator. The first author will randomize the participants before timepoint 0 (T0). A participant number corresponding with either the active condition or the sham condition determines the random allocation. In a situation where unblinding is permissible and to maintain the overall quality and legitimacy of the clinical trial, unblinding should occur only in exceptional circumstances when knowledge of the actual treatment is absolutely essential for further management and safety of the patient according to the Standard protocol items: recommendation for interventional trials (SPIRIT) statement [77]. Investigators are encouraged to discuss this with the Medical Ethical Review Committee of the Erasmus Medical Center (registration number 2018.065 – NL65209.078.18).

As a standard for effectiveness of the reduction in violent behavior, the results of a violence risk assessment instrument the Historical and Clinical Future Risk-assessment tool (HKT-R) [78] will be assessed before the intervention starts***.*** During the first session the self-reported measures will be assessed. EEG will additionally be performed during a resting-state task and during a passive-viewing empathy task [73]. The participants will then perform a rating viewing empathy task [73]. The patients will perform Point Subtraction Aggression Paradigm (PSAP) [79] to measure behavioral indices of the level of aggression.

After this, a series of ten tDCS sessions (two per day for five consecutive days) will follow. After every tDCS session the participants will fill in a questionnaire that assesses side effects. On Monday, after the last modulation on Friday, the patients will receive a post-intervention evaluation in which the rsEEG and the tasks (empathy task and PSAP task) again will be conducted. As a follow up, risk assessment scores of the participant will be obtained after 6 months to see whether risk reduction is maintained together with one last EEG session with the PSAP task.

### Overview of procedure

The procedures are as follows:

1. Inclusion of patients after informed consent.
2. Collection of information from patients on diagnosis, substance abuse, demographic information, and HKT-R.
3. Pre-intervention in which the patients undergo resting-state EEG (rsEEG) and perform the aggression task (PSAP) and the empathy task (victims of aggression). In addition, the patients will fill in the self-report questionnaires.
4. Intervention multichannel tDCS plus treatment as usual (TAU) or sham plus TAU: 20 min of anodal stimulation of the vmPFC and cathodal stimulation of the left supraorbital area with multichannel tDCS, two times daily for a period of five consecutive days.
5. Post-intervention where the patients undergo rsEEG and will perform the aggression task (PSAP) and the empathy task (victims of aggression), and fill out the self-report questionnaires again.
6. Outcome variables: the results of the aggression task and empathy task and the score on the HKT-R and the self-report questionnaires will be lower with respect to the results on the pre-test. Furthermore, we will investigate whether the functional connectivity (EEG) has changed at the post-test in contrast to the pre-test and if this differs within the two conditions (tDCS versus sham).
7. Follow up after 6 months where the patients will perform the aggression task and the empathy task one more time and the HKT-R will be administered.

**Fig. 2:**
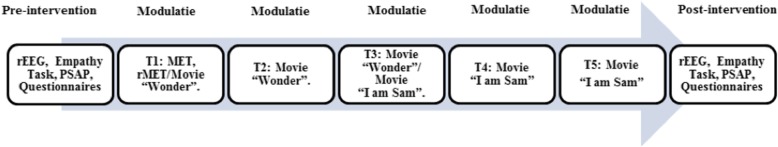
Flowchart of the trial. rsEEG, resting state electroencephalography, PSAP, Point Subtraction Aggression Paradigm, MET, Multifaceted Empathy Task, rMET, Reading the Mind in the Eyes Test, T, timepoint

**Fig. 3:**
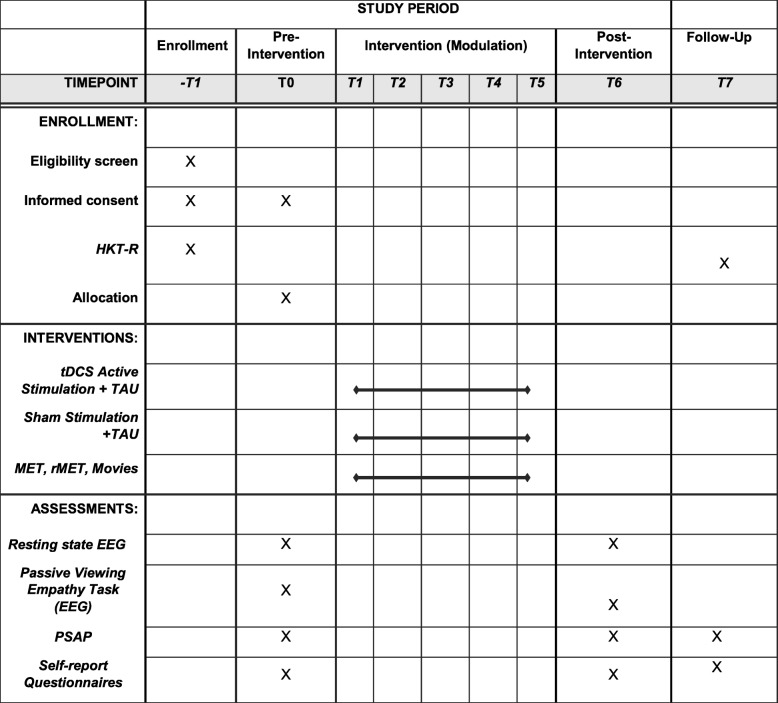
Standard protocol items: recommendation for interventional trials (SPIRIT) schedule of enrollment, interventions, and assessments. HKT-R, Dutch Risk Assessment Tool, PSAP, Point Subtraction Aggression Paradigm, MET, Multifaceted Empathy Task, rMET, Reading the Mind in the Eyes Test, EEG, electroencephalography TAU, treatment as usual

See Figs. 2 and 3 for a detailed overview of the procedure of the trial.

Multiple studies [80–83] have concluded that activating the brain state of the area of interest during stimulation, increases the effect of the modulation and contribute to optimize the intervention. An interesting study by Nissim and colleagues [84] demonstrated with fMRI that the optimal gains from using tDCS can be realized by simultaneously using behavior and modulation to stimulate neural networks. Therefore, to optimize our intervention and increase the activity of the vmPFC and the empathic abilities, the participants will be occupied with tasks and movies that trigger these brain states. On the first day, the participants will complete the Multifaceted Empathy Task (MET) [85] and the Reading the Mind in the Eyes test (rMEt) [86], to actively enhance their perspective and recognition of emotion. When finished, they will watch the movie “Wonder” (2017) for the first part of the stimulation week. For the remaining sessions they will watch the movie “I am Sam” (2001). The two movies enhance the empathic abilities and perspective, and therefore will contribute to the optimization of brain state.

### Sample

A total sample of 50 male patients with forensic substance abuse (25 active and 25 sham stimulation) will be tested in a double-blind placebo-controlled study, from which half of the patients will receive active stimulation plus TAU and the other half will receive a sham stimulation (placebo) plus TAU.

### Inclusion

In order to be eligible to participate in this study, a subject must meet all of the following criteria: male, age 18–60 years, good understanding of the Dutch language, diagnosed with an alcohol and/or cocaine SUD according to the *Diagnostic and statistical manual of mental disorders 5*^*th*^*edition* (DSM-5). The patients have to be abstinent and have an index offense in violence category listed in the HKT-R. These criteria have been selected due to the fact that men show more aggression on the PSAP task and in general show more violent behavior than women.

### Exclusion

Subjects meeting any of the following criteria will be excluded from participation in this study: major neurological conditions (e.g. traumatic brain injury) or major mental disorders (i.e. major depression, psychotic symptoms). Also, patients taking antipsychotic or other strong medication cannot participate in the study.

#### Recruitment

Recruitment will be active within the forensic institutions. Patients will be actively approached by the PhD student, or can sign up through a form at the department in which they receive their treatment. Research assistants will assist with the assessments. The entire research team will be trained extensively before they have an active role in the research.

After screening for exclusion criteria, the patients will receive more detailed information about the study and can decide whether to participate. An informed consent form will be signed prior to the actual test day.

#### Conditions

##### Experimental

The patients participating in the active, experimental condition start on Friday by completing the aforementioned self-report questionnaires. Also, EEG will be performed during a resting-state task (rsEEG) and during the passive viewing empathy task. After disconnecting the EEG device, participants will perform the empathy rating task and the PSAP. On Monday, the participants will start with the tDCS intervention; they will receive 20 min of tDCS two times each day. There will be a time interval of 3–4 h between the sessions, depending on the patients’ schedules. This will last for five consecutive days. One anodal electrode is placed on the position of Fpz and the other five cathodal electrodes are placed on AF3, Fz, AF4, F3, F4, according to the international 10–10 EEG system. The electrodes used are the Pistim EEG & tDCS hybrid electrodes with a 12-mm diameter.

##### Sham

The patients participating in the sham condition will follow exactly the same routine as the participants in the active condition except they will receive 2 mA for 30 s instead of the 20-min stimulation. This has proven to be effective for blinding as participants habituate to the sensation of stimulation within seconds of initiation of the current [87].

#### tDCS

For the intervention we will operate according to the Evidence-based guidelines on the therapeutic use of transcranial direct current stimulation (tDCS) [76]. Multichannel tDCS will be administered with a CE-certified neurostimulator (StarStim-8, NeuroElectrics) following the protocol [77]; the device is preprogrammed for stimulation with 2 mA for 20 min (experimental condition), or with 2 mA for 30 s (sham condition). We program the device for each participant, ensuring that the participant and investigator are blinded to the experimental condition. The experimental treatment is a 20-min tDCS session, two times a day for five consecutive days (ten sessions per participant). Multichannel tDCS will be applied over the vmPFC. The sham condition is the same as the experimental condition with the exception that there will only be a ramp up of the electrical stimulation to mimic the sensation of the stimulation.

##### Primary objective

The primary objective is to investigate tDCS as a new intervention to increase empathy and reduce violent behavior in offenders with substance abuse problems. This will be measured using the results of the empathy and aggression task from pre-test to post-test. In addition, we will study whether electrophysiological responses in the brain are affected by tDCS; this will be demonstrated through comparing the EEG from pre-test to post-test. We will use a mixed design to test whether event-related potential (ERP) amplitudes pre-post intervention differ between the active and sham group.

##### Secondary objective(s)

The secondary objective is to reduce craving in offenders with substance abuse problems. This will be assessed using the four self-report measurements on alcohol and drug craving, and to compare the results from pre-test to post-test. Other study parameters are the results from the self-report questionnaires. These will be compared with the other variables mentioned before and also compared from pre-test to post-test.

#### Instruments

##### Passive-viewing empathy task

To measure empathic abilities and how it changes between pre and post intervention, patients will participate in a passive-viewing empathy task. This measurement is based on previous research [72]. The pictures used in the passive-viewing task are selected based on ratings conducted through an anonymous online study (i.e. Amazon’s Mechanical Turk): 188 individuals participated in this study and each individual rated 45 pairs of pictures. These were aggression pictures and matched neutral pictures; both were rated on levels of arousal, aggressiveness, and valence. The study resulted in 40 pairs of pictures selected for the empathy tasks. The pictures display scenes with either two men, or one man and one woman aged between 20 and 25 years old. The men have a white complexion and the woman has a black skin tone. The majority of the aggression pictures (99%) had a male perpetrator and either a male or a female victim. The scenes involved physical, sexual, and verbal aggression. To control for stimulus-related confounding factors, all neutral pictures are carefully matched to the aggressive pictures. The neutral photographs were identical to the aggression-related photographs (pair-wise, i.e. the same persons, same location, same colors, same light), only without the aggressive action. Three types of pictures will be used in the experiment: (1) 40 pictures displaying an interaction between two individuals that is of a violent tone; (2) 40 pictures displaying an interaction between two individuals that is neutral, and (3) fifteen pictures displaying neutral objects like a bridge or a lamp (fillers). The fillers will not be used for further analyses: 95 pictures in total will be randomly presented for 6 s each, with intervals of 1.8 s between the pictures. Participants are instructed to look at each picture passively, because then the automatic neural response in the brain can be determined [72].

##### Rating empathy task [72]

Following the passive viewing empathy task, the pictures displaying the neutral and aggressive situations will be presented a second time. Participants are now instructed to rate the pictures by answering four questions. The first question is “Does this picture give you arousal?”. This is assessed to score arousal on a 9-point Likert scale (1 = no arousal to 9 = very high arousal). The following question was to assess the valence of the picture: “Does this give you a negative or a positive feeling?” This is assessed on a 9-point Likert scale (1 = negative emotions, 5 = no emotions, 9 = positive emotions). The last two questions assess the measure of state empathy. Last, there are two questions concerning empathy (i.e. measure of state empathy). One question assessed to what extent the participant could empathize with the perpetrator and the other question assessed to what extent the participants could empathize with the victim in the aggressive situations. Both questions will be scored on a 9-point Likert scale (1 = no empathy to 9 = high empathy). The total empathy task (passive viewing and rating) on average lasts 40 min [71].

##### The Point Subtraction Aggression Paradigm (PSAP)

Although multiple paradigms are used to measure aggression on an experimental manner in the laboratory, the PSAP task is known to be one of the best-validated instruments [88]. During the PSAP task participants play a game against a (fictive) opponent. The goal of the game is to earn more points than your opponent. Participants are offered 3 choices: (1) participants can earn points by pressing 100 times on the “1” button on the keyboard; (2) stealing points from your opponent by pressing ten times on the “2” button; or (3) guarding your own points as an escape so the opponent cannot steal from you by pressing ten times on the “3” button. When a participant picks option number 2 (aggressive response), the points that they steal will not add up as their own score, but will only be subtracted from the score of the opponent. If the (fictive) opponent is stealing points from the participants, this will be shown in red letters on the screen; in this way the participant will be “provoked”. If this provoking leads to the participant pressing the “2” (stealing) button more frequently, this can be seen as reactive aggression. When the participant is not provoked by this event, the aggression can be seen as proactive aggression. In this study the e-prime version of the PSAP consists of three 12-min sessions. The outcome of the PSAP, the aggressive response, is the number of “2” responses made by the participant. When the “2” response is a result of the provocation (the fictive opponent stealing points), then this indicates a reactive aggression response. If the “2” response is not a reaction to the provocation, then the reaction will be seen as proactive aggression response. Research [88] concludes that the PSAP has more ecological validity then for example the Taylor Aggression Paradigm (TAP) [89]. This is due to the following advantages: first, the PSAP task offers the participant an option to actively withdraw (pressing the “3” key), something that is not included in other paradigm tasks. Second, in the PSAP task the aggressive response (pressing the “2” button) is not receiving points and therefore is more ecologically valid then other tasks that do not have this option. An aggressive reaction is a cost-benefit consideration, so choosing an option to steal points that will not add up to your own score for winning could be considered as a costly option.

#### Self-report questionnaires

##### Self-Report Psychopathy Scale Short-Form (SRP-SF)

We use the Dutch short version of the SRP-SF [90] in order to assess psychopathic traits. The SRP-SF consists of a subset of 29 of the 64 original items and is a self-report questionnaire in which participants are asked to rate statements using a 5-point Likert scale. The questionnaire consists of four subscales: interpersonal manipulation (manipulation and deception), callous affect (lack of empathy or regret), instable lifestyle (impulsivity and sensation-seeking behavior) and criminal behavior (delinquency and criminal behavior).

##### Reactive and Proactive Aggression Questionnaire (RPQ)

We use the Dutch version of the RPQ [91] in order to assess aggression. The RPQ is a 23-item self-report questionnaire in which the participant has to give a rating based on how often this behavior has occurred in the past, on a 3-point scale (“never”, “sometimes”, or “often”). Next to an overall total aggression score, the test provides two separate measures of proactive aggression (deliberate and planned aggression) and reactive aggression (aggression as a reaction to an unplanned circumstance).

##### Interpersonal Reactivity Index (IRI)

We use the Dutch version of the IRI [92] in order to assess empathy. This is a commonly used self-report instrument designed to assess empathic tendencies. The IRI consists of four separate subscales: perspective taking (PT), fantasy (FS), empathic concern (EC), and personal distress (PD).

##### Toronto Alexithymia Scale (TAS-20)

We use the Dutch version of the TAS-20 [93] in order to assess alexithymia. The TAS-20 is a self-report scale comprising 20 items. Each item is rated on a 5-point Likert scale ranging from 1 (strongly disagree) to 5 (strongly agree). The TAS20 is a reliable and valid measure of emotion processing in adults that includes a total score and three subscales: difficulty identifying feelings (DIF), difficulty describing feelings (DDF), and externally-oriented thinking (EOT).

##### Risky, Impulsive, and Self-Destructive Behavior Questionnaire (RISQ)

We use the Dutch version of the RISQ 96] in order to assess risky and impulsive behavior. The RISQ is a 38-item self-report questionnaire-based measure, assessing eight domain-specific factors (measuring drug use, aggression, self-harm, gambling, risky sexual behavior, impulsive eating, heavy alcohol use, and reckless behavior).

##### Behavioral Impulsivity Scale (BIS-11) [94]

We use the Dutch version of the BIS-11 [94] in order to assess the personality and behavioral construct of impulsivity. The BIS-11 is a questionnaire that consists of one of the three second-order facets of impulsivity. The 30-item self-report questionnaire consists of six subscales: attention (i.e. focusing on current tasks), cognitive instability (i.e. intruding thoughts), motor impulsiveness (i.e. acting quickly), perseverance (stable lifestyle), cognitive complexity (i.e. enjoys mental challenges), and self-control, (i.e. plans and thinks deliberatively).

##### Alcohol Use Disorders Identification Test (AUDIT)

We use the Dutch version of the AUDIT [95] in order to assess alcohol use. The 10-item AUDIT includes questions to assess alcohol intake (questions 1–3), alcohol dependence (questions 4–6), and alcohol-related problems (questions 7–10). Questions 1–8 are scored from 0 to 4, questions 9 and 10 are scored 0, 2, or 4, resulting in a maximum AUDIT score of 40.

##### Drug Use Disorder Identification Test (DUDIT)

We use the Dutch version of the DUDIT [96] in order to assess drug use. The DUDIT is an 11-item screening instrument to assess non-alcohol drug use patterns and various drug-related problems. The first nine items are assessed on a 5-point Likert scale and the last two items are scored on a 3-point scale. Higher scores suggest more severe drug problems.

##### Obsessive Compulsive Drug Use Scale (OC-DUS-version Cocaine)

We use the Dutch version of the OC-DUS [97] in order to assess drug craving. The OC-DUS version Cocaine is a 13-item self-report questionnaire that assesses the inability to control or resist cocaine-related thoughts and behaviors, frequency and impact of thoughts and impulses related to cocaine use, and the degree of interference.

##### Obsessive Compulsive Drinking Scale (OCDS)

We use the Dutch version of the OCDS [98] in order to assess alcohol craving. The 22-item Dutch version of the OCDS was developed to reflect obsession and compulsivity related to craving and drinking behavior. The OCDS has been shown to be specific for the obsessive and compulsive characteristics of drinking-related thought, urges to drink, and the ability to resist those thoughts and urges in alcohol-abusing and alcohol-dependent individuals.

### Outcomes

#### Primary outcomes

The primary outcomes are empathic abilities, aggressive behavior, and the electrophysiological response in the brain. Empathic abilities will be measured using the Passive Viewing Empathy Task [72], followed by the Rating Empathy Task [72]. The Passive Viewing Empathy task examines the electrophysiological outcomes of empathic processing while observing aggressive situations. The results will be measured in the amplitude of specific ERPs. The early ERP component resembles the P300, which is a positive voltage in the latency of 300–650 ms and the late ERP component reflects the late positive potential (LPP), a sustained positive potential identified at around 400–1000 ms. Specifically, P3 and LPP appear to be a measure of empathic processing, and therefore make EEG an adequate tool to indicate any change in empathic abilities, reflected as a change in the amplitude of the ERPs [73]. In the Rating Empathy Task [72] the pictures displaying the neutral and aggressive situations will be presented a second time. Participants are now instructed to rate the pictures by answering four questions that will result in an outcome in emotional valence, empathy for the perpetrator, empathy for the victim, and arousal. The outcome of the self-reported assessment of empathy is used to measure state empathy.

Aggressive behavior will be assessed using the PSAP task. The PSAP task is known to be one of the best-validated instruments in provoking aggression in the laboratory [88]. In this task the participants will be “provoked” by a (fictive) opponent who will steal points from them. If the (fictive) opponent is stealing points from the participants, this will be shown in red letters on the screen. If this provoking leads to the participant pressing the “2” (stealing) button more frequently, this can be seen as reactive aggression. The results of the PSAP task will be the amount of pressing the “2” button in proportion to the total amount of pressing the buttons.

As a standard for effectiveness of the reduction of violent behavior next to the PSAP, the results of a violence risk assessment instrument (HKT-R) [78] will be assessed before the intervention starts. The HKT-R is one of the mandatory risk-assessment tools in forensic institutions. The clinical, historical, and future indicators of violent behavior and the risk of recidivism will be used as the outcome for the reduction of violent behavior.

Functional brain changes will be measured using rsEEG, with a resting-state task to measure the baseline of the brain activity of the participant in a resting-state condition and the passive viewing empathy task to measure the electrophysiological changes in brain function during empathic induction caused by the intervention. The expectation is that patients who receive tDCS will show higher event-related potential (ERP) towards the pictures of the victims after the intervention compared with patients who have receive the sham condition.

After 6 months, as a standard for effectiveness of the reduction of violent behavior, the results of the aggression task (PSAP) and the risk-assessment tool (HKT-R) will be compared to the results obtained before the intervention. The HKT-R risk level will be obtained to see whether there is a longitudinal effect of tDCS on aggression and risk reduction. In addition, to check whether the effect of the tDCS intervention is long-lasting, the participants will complete the self-report questionnaires, the empathy task, and the aggression task once more.

#### Secondary outcomes

Beside the primary outcomes, the current study distinguishes multiple secondary outcomes. These will be measured using the self-report questionnaires described in the 'Instruments' section. All the secondary outcomes will be assessed during T0 and again at T6.

### Statistical analysis

Different general linear models (GLM) in SPSS will be used to analyze the main parameters. For instance, analyzing the empathy and aggression outcome (empathy ratings and b responses in the PSAP task), the outcome variables will be handled as the dependent variables. In addition, the group variable (active versus sham) will be used as the independent between-group variable, whereas the pre-post time will be included as a within-subject independent variable. A mixed design will be used in which the pre-post intervention in the experimental group will be compared with the pre-post intervention aggression outcomes in the sham group. The mixed design will be used for the PSAP outcomes, empathy ratings, self-report questionnaires, and assessment of risk of violence with the HKT-R instrument.

### Power analysis

The sample size is based on the primary outcome and other studies from the aforementioned guidelines [76] and seen as achievable. The sample size is based on research [100] that reported large effect sizes (i.e. partial eta squared 0.25 and 0.21) for the active versus sham condition, using 15 participants per condition for three conditions. Because we have two experimental conditions but also include covariates, a sample size of 50 subjects (25 per condition) is considered to have enough power to detect an effect, when power is set to 80% and alpha of 5%, two sided.

## Discussion

This study protocol describes the design of an intervention with multichannel tDCS targeting bilateral vmPFC next to TAU, in comparison with the sham condition and TAU. This study will explore the opportunities to introduce a new intervention to improve empathy, reduce violence, and reduce craving in substance-use offenders.

The present study has several strengths. First, to our knowledge only a few studies focus on increasing empathic abilities [62–67, 101] or modulate externalizing behavior [42, 68–70], but none of the studies focus on the implication of tDCS in a forensic sample. These individuals are in need of effective care, and by modulating the brain activity this can be a first step towards a new treatment program.

Second, tDCS may also influence substance abuse [49–61] and, in turn, influence the relationship between substance abuse and violent behavior. This could lead to a decrease in recidivism in forensic institutions.

Third, we will use a wide range of instruments and will gather information through multiple sources. We will obtain rsEEG, questionnaires about psychopathic traits (SRP-SF) [90], aggression (RPQ) [91], empathy (IRI) [92], alexithymia (TAS-20) [93], risky and impulsive behavior (RISQ) [99], and impulsivity (BISS11) [94]. For measuring substance abuse the study includes questionnaires that focus on alcohol use (AUDIT) [95], alcohol craving (OCDS) [96], drug use (DUDIT) [97], and drug craving (OC-DUS-version Cocaine) [98]. In addition, we have the results on the PSAP and empathy task.

Moreover, we will also include the participants who drop out during the intervention in our 5-day intervention program. This will give insight into the consequences of the formal procedures preceding the intervention and may eventually enable us to describe profiles of the intervention with tDCS in treatment success and failure.

Finally, this study will contribute to development of a cheaper and less invasive treatment for substance abuse. As mentioned before, problems that patients with substance abuse bring an enormous burden to the community (financial and safety). Previous research has found that substance abuse, especially alcohol and cocaine, are related to (violent) criminal behavior. Money invested in treatment may lead to a large reduction in the costs associated with substance abuse. Nevertheless, current interventions seem insufficient in the treatment of substance abuse in forensic mental health care and are not sufficient enough to reduce the risk of violence risk; 66% of these patients reoffend.

Despite the strengths of this current study, several limitations may threaten the quality of our study. The greatest challenge will be to have an adequate response rate at baseline and at the end of the 5-day treatment. Our target group will be conducted in a forensic institution and is participation is voluntary, so the participants can decide to quit at any time. The population has an extensive history of treatment and criminal justice, so they might have negative experiences with treatment or research studies. Moreover, the participants already have filled in numerous questionnaires in their time spent in institutions, such that they may have become tired or suspicious about the purpose of the proposed study. To keep the nonresponse rate as low as possible, the researchers conducting this study are trained to motivate and encourage the participants to complete the program. The interaction will be transparent, attentive, and flexible and the researchers will be prepared to give clear information about the purpose, goal, and aim of the current study at any time. Furthermore, a treatment that uses neurostimulation and EEG might seem scary and new to the participants as compared to TAU, so extra explanation might be needed to assure the participants that it is safe. So, to recruit as many participants as we aim to in this study will require a high degree of motivation.

Finally, an active control group (i.e. sham modulation and TAU) might lead to smaller effect sizes with active tDCS than when the tDCS treatment is compared to no treatment or a wait-list group. However, considering the seriousness of the problems of the forensic substance use offenders it would not be ethical to let them wait for an intervention and it remains important to get a sense of the effects of placebo versus the effects of active stimulation.

In conclusion, with the present study design we are able to explore what the added effectiveness is of tDCS plus TAU in comparison to sham plus TAU, which could provide valuable information for institutions, researchers, psychologists, and the professionals in the field of criminal justice. In addition, this study may help to reduce craving in forensic substance use offenders and thus reduce not only violent behavior but also recidivism. The study will contribute to knowledge about increasing empathic abilities and the functioning of the vmPFC. Specifically, this study may give insight into implementing tDCS on the vmPFC in the TAU and the setting of daily clinical practice in this complex, multiple problem target group.

## Trial status

The current study started in February 2019. The data collection will run until the 50 participants all completed the 5-day program. Follow up will be conducted after 6 months to test whether the effects of the intervention are still present. Until then, the intervention effects are unknown.

## Data Availability

Not applicable.

## Abbreviations

ACC: Anterior cingulate cortex
AIC: Anterior insular cortex
AUDIT: Alcohol Use Disorder Identification Test
BIS11: Behavioral Impulsivity Scale
CCMO: Central Committee on Research Involving Human Subjects (*Central Commissie Mensgebonden Onderzoek*)
DSM-5: Diagnostic and statistical manual of mental disorders
DUDIT: Drug Use Disorder Identification Test
EEG: Electroencephalography
ERP: Event-related potential
EudraCT: European Union Drug Regulating Authorities Clinical Trials database
fMRI: Functional magnetic resonance imaging
GCP: Good Clinical Practice
HKT-R: Dutch Risk Assessment Tool
IB: Investigator’s brochure
IC: Informed consent
IMP: Investigational medicinal product
IMPD: Investigational medicinal product dossier
IRI: Interpersonal Reactivity Index
LTP: Long-term potentiation
MET: Multifaceted Empathy Task
METC: Medical Research Ethics Committee (MREC) (*Medische Ethische Toetsings Commissie* (METC))
OCDS: Obsessive Compulsive Drinking Scale
OCDUS: Obsessive Compulsive Drug Use Scale
PFC: Prefrontal cortex
PSAP: Point Subtraction Aggression Paradigm
RISQ: Risky, Impulsive, and Self-Destructive Behavior Questionnaire
rMET: Reading the Mind in the Eyes Test
RPQ: Reactive and Proactive Aggression Questionnaire
rsEEG: Resting-state electroencephalography
(S)AE: (Serious) adverse event
SPC: Summary of product characteristics (Officiële Productinformatie Sponsor): the sponsor is the party that commissions the organization or performance of the research, for example a pharmaceutical company, academic hospital, scientific organization or investigator. A party that provides funding for a study but does not commission it is not regarded as the sponsor, but referred to as a subsidiary party
SPIRIT: Standard protocol items: recommendation for interventional trials
SRP-SF: Self-Report Psychopathy Scale Short Form
SUD: Substance abuse disorder
SUSAR: Suspected unsuspected serious adverse reaction
TAU: Treatment as usual
tDCS: Transcranial direct current stimulation
vmPFC: Ventromedial prefrontal cortex
Wbp: Personal Data Protection Act (*Wet Bescherming Persoonsgegevens*)
WMO: Medical Research Involving Human Subjects act (*Wet Medisch-wetenschappelijk Onderzoek met mensen*)
